# Professor Valentin's Physiology
*A Text-Book of Physiology. By Dr. G. Valentin, Professor of Physiology in the University of Berne. Translated and Edited from the third German edition, by William Brinton, M.D., Licentiate of the Royal College of Physicians. London: Renshaw. 1853.


**Published:** 1854-01-01

**Authors:** 


### ART. VIII.—PROFESSOR VALENTIN'S PHYSIOLOGY.*

THE "LEHRBUCH DER PHYSIOLOGIE," by Dr. Valentin, Professor of Physiology in the University of Berne, is an elaborate systematic treatise on physiology, which enjoys a very high reputation in the medical schools of Germany; we are therefore under great obligation to Dr. Brinton for having, in the form of an abridgment, rendered the substance of so valuable a work accessible to the English student. We have, it is true, many excellent elementary works on physiology—Todd and Bowman, Carpenter, Kirke, Mayo, Milligan's translation of Magendie, &c.,—but the present text-book has many attractive features which cannot fail to interest both the senior and junior members of the profession. We have, in carefully perusing the volume, been struck with the extreme perspicuity of the style, the vast amount of information condensed into a narrow compass, and the clear explanation which is given in describing successively the different organic functions of the animal economy. We have also been interested in reading the opinions of so high an authority as Professor Valentin on many doubtful and obscure points in physiology. "*Quot homines tot sententiæ.*" Since the days of Haller—but we may go back to a remoter age—theories of organization, life, digestion, absorption, animal heat, respiration, nutrition, muscular action, and innervation, have flitted across the horizon of medical science, one fascinating theory eclipsing another as rapidly as new observations have revealed to us new discoveries. Looking back to the period when Cabanis published his sketches of the "Revolutions of Medical Science," what changes have not occurred in our physiological and pathological views? but we must not pause to moralize, "*tempora mutantur et nos mutantur in illis,*" progression is the order of the day, and unceasing change in all things the great law of nature.

The "Text-Book of Physiology" before us is a goodly-sized volume,

* A Text-Book of Physiology. By Dr. G. Valentin, Professor of Physiology in the University of Berne. Translated and Edited from the third German edition, by William Brinton, M.D., Licentiate of the Royal College of Physicians. London: Renshaw. 1853.

extending to 684 pages; it is divided into nineteen chapters, the contents of which are arranged in numerical sections, any one of which, with the assistance of a good index at the end of the book, may be referred to with great facility. The chapters beginning with one on "Organization and Life," treat successively, as we find in other elementary works on physiology, of the functions of all the different organs of the body; but as connected with our own speciality we prefer restricting our attention to the section which refers more particularly to the functions of the nervous system. We were, indeed, as we had premised, desirous of ascertaining Professor Valentin's views upon many interesting points, such as the correlation supposed to exist between the size of the cerebrum or number of its convolutions, and the manifestation of the intellect, the functions of the cerebellum, &c. Upon the former subject Professor Valentin remarks—

"There is no doubt that an abnormal smallness of the brain is connected with idiocy. And it is very probable that persons distinguished for their intellectual powers possess brains which are either as a whole or in particular parts. But it is far more difficult to prove this excess than the converse diminution *for the abnormal circumstances which precede death may themselves produce a deceptive increase of weight;* and we have a right to suppose that the mental endowments are materially influenced, not merely by the quantity of the organ, but also by its quality and relative capacity." (*Sect.* 2059, p. 602.)

There is, we believe, much truth in this observation. Among pseudomorbid appearances which occur after death, it is very possible there may be sometimes an increase of weight, owing to the exsudation of a large quantity of serum in the ventricles. In the act of dying effusion certainly may go on very rapidly.

"The high forehead" continues the Professor, "which is frequently regarded as an external indication of mental power, certainly does generally depend on a greater development of the anterior lobes, and of those parts of the skull which cover them. Still, this does not justify the conclusion that it is those segments of the cerebral mass which exclusively regulate the higher mental capacities or many of the faculties that express them—such as, for instance, eloquence. Comparative and pathological anatomy unite to testify that the middle and posterior lobes of the brain, and many of its internal swellings (such as the pes hippocampi and pes accessorius which lie in the posterior corner of the lateral ventricle of each hemisphere) are at least as important as its anterior segments." (*Ibid.*)

It may be remembered that Desmoulins suggested the theory of there being a correlation between the higher manifestations of the intellect and the number and depth, or rather the extent of surface of the cerebral convolutions.

"It has often been maintained," observes the Professor, "that in men distinguished for intellect the convolutions of the two cerebral hemispheres are more numerous and less symmetrical. But the fact itself is by no means established. And besides this, experience teaches that the advantages which are perhaps associated with the convoluted arrangement, may be quite annihilated by internal disease The *brain of a cretin often exhibits large and complicated convolutions*, while its cavities are distended by copious fluid exsudations." (*Sect.* 2060, p. 602.)

The late Dugald Stewart in commenting upon and censuring the hypothetical doctrines of Hartley, Darwin, and Priestley, seems to hazard a prophecy that no physiological researches will ever throw any light on the connexion which exists between the mind and the body. "I object," he says, "to such investigations, as being merely a waste of labour and ingenuity on questions to which the human faculties are altogether incompetent." ("Preliminary Dissertation to the Philosophical Essays.") But had Dugald Stewart lived to see the recent progress of physiology and pathology, and the method of observation now pursued, he would probably have modified in some measure so disparaging a prediction. The theory of vibrations was a sheer hypothesis; unsupported by observation, it started fully formed into existence, like Pallas ready armed out of the head of Minerva; but the connexion which is now sought to be established between physiology and psychology proceeds, we venture to affirm, upon more philosophical principles—upon inductive observation, rather than pure speculation. One chief cause of the little assistance physiology renders to psychology is very truly attributed by Professor Valentin to our "deficient knowedge of the physiology of the nervous centre," and another, he suggests, may be ascribed to the way in which we conceive the mental functions to take place.

"We describe," he observes, "certain external phenomena as results of knowledge, judgment, or reason, without recollecting that the basis here assumed to be an unit is the result of a series of links to which we have no access. We have seen that the perception of the simplest sensuous impression, and the excitation of the slightest muscular movement, depend upon a transfer and conduction of certain material changes which mutually conditionate each other, and play into one another like the cogwheels of a machine. Hence we are justified in conjecturing, that what is apparently the most direct mental action also proceeds from a series of mutual tensions and transfers, and that the failure of any link of the chain leads to an error or false conclusion. The general significance of such processes may be easily imagined from the analysis offered by these simple phenomena. But the satisfactory investigation of their details, the only true self-knowledge, will probably for ever baffle the spirit of human inquiry." (*Sect.* 2068, p. 604.)

Let us hope that this dark foreboding of the learned Professor may not be verified; we recognise the difficulties which embarrass our investigations only in the hope of surmounting them; we still look for progression, and at all events conceive that no one has a right either in mental or physical science, to say "thus far shalt thou go and no farther;" a sceptical philosopher may, like Canute the Great, plant his chair at the margin of the sea to reprove his disciples, when the waves appear shining tranquilly at their lowest ebb, but ere long he will find the tide rise, and the accumulating waters swelling round him will compel him to retire from his position. The progression of human knowledge is thus irresistible—he, however, who would assist in extending its boundaries, in accelerating its current, must not be restrained by any circumscribed ideas of *finality;* he must embark with a cheerful faith upon all his investigations in the full assurance that truth will unveil herself to all who diligently seek her, and that she has imposed no restriction upon our footsteps.

To return: the functions of the cerebellum have been the subject of much discussion; some physiologists from the experiments made on living animals have inferred that it is the organ which co-ordinates the muscular movements of the body. But however plausible this view may be, the unsteady movements of animals—birds, for example—whose cerebellum has been removed, and the ineffective flutterings which accompany them, may, it is surmised by Professor Valentin, be rather consequent upon certain local conditions of progression or flight—such, for instance, as the proper fixation and adjustment of the spinal column. The idea that the cerebellum has any special influence over the sexual organs, the Professor emphatically repudiates.

"Experience," he says, "does not confirm this theory. It is true that the seminal ducts, the oviducts, and the uterus of the domestic mammalia may be thrown into contraction from the cerebellum. But the same effect can be produced through other parts of the nervous centres (lesion of the spinal cord or medulla oblongata). The cerebellum of geldings is as large as that of stallions, and Flourens found that a cock from whom this part of the nervous centre had been removed still made distinct attempts at copulation." (*Sect.* 2047, p. 599.)

The Professor further remarks that—

"Degeneration of the human cerebellum does not necessarily give rise to imbecility or any other affection of the mental powers. Destruction of one of its hemispheres has sometimes been accompanied by an uncertainty of gait, a tendency to rotatory movements or hemiplegia, usually of the opposite side, but limited disease of the cerebellum may exist without any considerable disturbance of the action of the voluntary muscles." (*Sect.* 2048, p. 600.)

We had marked several other passages for extract, but with much reluctance must, at all events for the present, forbear transcribing them; almost every section in this abridgment of Valentin's great work on physiology, might indeed suggest a running commentary. The translation has been very carefully and faithfully, and we may add elegantly, rendered; and unlike translations in general from the German, its style is fluent and agreeable. We should add that the diagrams and illustrations, above three hundred in number, are beautifully executed and admirably represent the subjects delineated. It is evident, indeed, that Dr. Brinton has bestowed very great pains on the volume before us; his critical knowledge of the German language well qualified him to execute a task which we can only conceive his having undertaken as a "work of love;" and we have no hesitation in expressing our conviction that this "Text-Book of Physiology" deserves to be as popular in our medical schools as the original is in Germany. We therefore cordially recommend it to the students at our English universities.

---

